# SettleIN: Using a Manualised Intervention to Facilitate the Adjustment of Older Adults with Dementia Following Placement into Residential Care

**DOI:** 10.3390/ijerph17072606

**Published:** 2020-04-10

**Authors:** Caroline A Saint-Bryant, Judy Murrill, Janine K Hayward, Kayleigh-Marie Nunez, Aimee Spector

**Affiliations:** 1 Research Department of Clinical, Educational and Health Psychology, University College London, 1-19 Torrington Place, London WC1E 7HB, UK; judy.murrill.13@ucl.ac.uk (J.M.); janine.hayward.13@ucl.ac.uk (J.K.H.); a.spector@ucl.ac.uk (A.S.); 2 Wolfson Centre for Age Related Diseases, Kings College London, Wolfson Wing, Hodgkin Building, Guys Campus, London SE1 1UL, UK; Kayleigh-marie.nunez@kcl.ac.uk

**Keywords:** dementia, adjustment, residential care, psychological wellbeing, staff training, quality of life

## Abstract

The authors examined the feasibility of delivering an adapted version of SettleIN, a manualised staff-led programme designed to facilitate adjustment to care for new residents with dementia. The effects of SettleIN on resident adjustment, mood and quality of life were also investigated. A pilot randomised controlled trial was conducted. Nineteen new residents with dementia and 21 staff participants were recruited. Residents were randomly assigned to receive the SettleIN programme or residential care as usual. Resident quality of life, mood and overall adjustment were measured at baseline and post-intervention, in week seven. Interviews were conducted with staff in week seven to explore intervention feasibility. Despite medium to large effect sizes, there was no significant difference in mean change scores between the two conditions, with regards to quality of life, psychological wellbeing or overall adjustment outcomes. Qualitative feedback indicated that SettleIN was not feasible across all areas, with problems around recruitment and practicality. However, SettleIN was deemed feasible in terms of retention and acceptability among staff. The majority of staff felt that SettleIN was beneficial for residents but that organisational and programme factors impacted upon intervention feasibility. Further exploration of organisational barriers is needed in order to reduce the impact of such factors on care home research.

## 1. Introduction

Sixty-nine percent of people with dementia live in a residential care setting [1]. The circumstances around relocating into care can mean that the transition is rushed. Many residents consequently feel powerless in the decision to move and experience negative outcomes, including increased emotional responses and difficulties adjusting [2].

Relocating into residential care has been linked to increased cognitive decline, behavioural and psychological symptoms of dementia (BPSD) [3]. One study found that 34.3 percent of residents who relocated from their own home into residential care met criteria for depression symptomology [4]. Residents have also reported having a poorer quality of life following relocation [5]. This finding, however, has not been consistently supported, suggesting that successful adjustment is possible [6].

To successfully adjust, people with dementia need to accomplish three processes: settle in, fit in and find meaning within this process [7]. To achieve this, they must adjust to the schedule of the home, form new meaningful relationships and modify their identity as they adapt. A review of the research also focused on the specific factors affecting relocation adjustment [3]. Sury and colleagues [3] highlighted the role of resident autonomy, the physical environment, relationships, sociocultural needs and stimulating activity. A primary recommendation of the review was that an intervention, which considered these factors, needed to be developed to aid resident adjustment. Various strategies were recommended as part of the proposed intervention, including creating a home-like environment and having a buddy system in place.

Care home staff are considered to have a vital role in the transition process [8]. Their position allows them to promote new relationships among people with dementia. Staff training can therefore be a means of reflecting with staff on the emotional impact of relocation and ensuring that suggested strategies are incorporated into everyday care [8].

In response to this unmet need for resident adaptation support and related staff training during relocation, Hayward, Nunez, Ballard and Spector [9] created the SettleIN programme. SettleIN is a person-centred tool for people with dementia that is designed to facilitate healthy adjustment. Hayward et al.’s feasibility, pre- and post-intervention pilot study was conducted (N = 13) in order to evaluate the acceptability of SettleIN and the effectiveness of the programme in improving residents’ mood and quality of life. In its existing form, SettleIN was not found to be feasible to deliver in care homes across the UK and, due to high attrition rates of 62 percent, the study lacked sufficient data to draw conclusions on programme effectiveness. The SettleIN programme was found to be highly acceptable among stakeholders and staff who implemented the programme [9]; this is key for an effective intervention [10].

Since Hayward’s initial pilot study, there appears to be no further published interventions to facilitate resident adjustment [11] but recent research continues to conclude that an intervention of this nature is needed [12]. The main aim of this study was therefore to create a more feasible, enhanced version of SettleIN. Recommendations for improvement by Hayward and colleagues were adopted in a second feasibility study. Recommendations included reducing and simplifying SettleIN, as well as removing dependence on family members for programme completion. For more information about these recommendations, see Hayward et al. [9]. This study also expanded on the research carried out by Hayward by including a control group, enabling comparison to natural adjustment.

In the form of a pilot randomised controlled trial, the study examined the feasibility and effectiveness of the enhanced version of SettleIN. It was hypothesised that (a) those receiving SettleIN would experience an improvement in their mood and increase in their quality of life compared to those in the control group, and (b) SettleIN would be feasible for staff.

## 2. Method

### 2.1. Phase One: Developing the Intervention

The framework of designing, delivering and evaluating interventions is often not linear [10]. Drawing upon feedback from the previous study, this study returned to the development phase to modify the programme. Modifications made included reducing the intensity of SettleIN, formalising staff supervision, removing dependency on family members and adding an additional module for residents who struggled to engage.

#### Consultation 

Seven care homes involved in Hayward’s trial were invited to discuss the changes made to the programme. Due to high levels of staff turnover, only two staff members from one home were available to participate. Both staff members were care assistants who had delivered the programme in the previous trial.

The principal researchers met with the care assistants individually for approximately forty-five minutes. They were shown the enhanced SettleIN programme, following which the principal researchers conducted a semi-structured interview. See Table 1 for a summary of responses. Following this consultation, further changes to the new SettleIN workbook were finalised and made ready for use in the feasibility study.

### 2.2. Phase Two: Feasibility Study of the Enhanced SettleIN Intervention

#### 2.2.1. Design 

The study used a between-subjects randomised experimental design to evaluate the feasibility of implementing an enhanced version of SettleIN. The study also focused on the effects of the SettleIN programme on new residents’ quality of life, psychological wellbeing and overall adjustment. A sequential explanatory mixed-methods design was employed with quantitative results collected and qualitative data then used to build on the quantitative findings.

#### 2.2.2. Ethical approval

Ethical approval, covering both phases, was obtained from both University College London Joint Research Office and the Camden and Kings Cross Research Ethics Committee (Ref: 15/LO/0611).

#### 2.2.3. Recruitment

##### Setting 

Between April 2017 and January 2018, 156 care homes were contacted to take part in the research. Care homes were identified using the Care Quality Commission (2013) care directory and the Enabling Research in Care Homes (ENRICH) database. Opportunity sampling was also employed.

Of the 156 care homes initially contacted, 10 care homes responded expressing an interest in participation. The care homes that did not respond were contacted again by the principal researchers. From this an additional 17 care home managers expressed an interest in partaking in the research.

The principal researchers met with the care home managers from the 27 homes to clarify eligibility (see Table 2) and provide information about SettleIN. From this, formal written consent was obtained from 17 care homes. All care homes were offered a certificate for partaking in the research.

##### Participants

Using G*Power 3 [13], it was calculated that a minimum of 24 resident participants would be required to achieve sufficient power (0.8) at a 0.05 level of statistical significance and to detect a conservative effect size of 0.3, which is typical for a pilot study and chosen due to the lack of methodologically equivalent research. To account for possible attrition, the study aimed for a sample size of 30. However, as this was a pilot study, the chief aim was to assess feasibility for a full trial, retention rates and effect sizes.

In line with recent evidence about the importance of managerial support [14], a partnership approach was emphasised with recruited care homes. Participating managers agreed to take a key role in the running of SettleIN. They were encouraged to talk to new residents and carers about SettleIN as part of their routine process when discussing relocation. The researchers then assessed resident suitability and sought formal consent.

The intervention was a staff-led programme which required one or two staff participants for every resident participant. Care home managers provided staff members with information leaflets about SettleIN. The principal researchers then gained formal consent. All staff participants were given a £10 high street gift voucher and a certificate for partaking in the research.

#### 2.2.4. Procedure 

Once written consent was obtained from the staff participant, and the resident (or their family), the baseline assessment was conducted.

##### Randomisation 

Following baseline assessment, each resident was randomised to one of two conditions: the intervention group, which received the SettleIN programme, or the control group, which received residential care as usual. Care as usual consisted of the existing standard practice and adjustment support given by the homes. This was monitored as part of the demographic measures taken. An independent researcher randomised participants using a computer-generated sequencing programme. Block Randomisation was employed using a fixed block size of four to ensure an equal proportion of residents in each condition. The researcher responsible for data collection remained blind to the condition, ensuring that the study was single blinded.

##### Intervention Training

Staff participants, working with residents assigned to the intervention condition, attended a one to one training session on the SettleIN programme, conducted by a principal researcher at the care home and lasting one hour and 15 min.

The training involved an introduction to the factors that influence successful adjustment. The training systematically went through each module of the programme and covered how to deliver the tasks within modules. Staff in the control condition did not receive training.

##### The SettleIN Programme

The SettleIN programme is a staff-led manualised intervention that consists of four mandatory modules: orientation, lifestyle, friends and family and identity, along with one optional module: for residents who struggle to engage. The modules consist of various activities that are designed to promote healthy adjustment in new residents (see Table 3 for example module questions).

All of the activities were carried out with the residents by staff participants, normally a resident’s key worker. Following activity completion, the staff participant was required to document the relevant information in the workbook. The programme was designed to take a full time staff member four weeks to complete, taking up to six weeks for part time staff.

Staff were offered weekly telephone supervision, which lasted an average of 10 min. This focused on the challenges experienced and gave staff the opportunity to share positive experiences. Supervision involved problem solving difficulties, including liaising with management to review their support.

#### 2.2.5. Measures 

Measures were collected from all residents and staff participants at two stages: baseline (week zero) and post-intervention (week seven). The functional stage of dementia and demographic information were collected at baseline only. In week seven, 30 min interviews were conducted with the staff participants who had received training and the SettleIN workbooks were collected to provide information on implementation.

##### Demographics

Information regarding resident demographics and relevant medical information was obtained from residents’ care plans. Staff demographics, usual care home adjustment support (such as an orientation programme and any procedures to keep families informed), and resident adjustment support (including prior visits to the home) were also asked about.

##### Functional Stage of Dementia

The tool, completed with staff, consists of seven main stages from normal functioning (stage one) to severe dementia (stage seven), with five substages at stage six and six substages at stage seven [15]. The FAST has been found to be both a reliable and valid assessment tool across all stages of dementia severity [16].

##### Quality of Life

Quality of life was measured using the Quality of Life in Alzheimer’s disease (QOL-AD) [17]. This 13 item measure is rated on a 4-point scale, with answers ranging from poor (1) to excellent (4). A total score is calculated, ranging from 13 to 52, with a higher total score suggesting a higher quality of life. The measure consists of the following dimensions: finances, physical health, mental health and social activities. The QOL-AD was completed by both the resident, where possible, and their keyworker. The measure has high levels of internal consistency for people with dementia (Cronbach’s alpha = 0.84) and by proxies (Cronbach’s alpha = 0.86) [18].

##### Psychological Wellbeing

The Cornell Scale for Depression in Dementia (CSDD) [19] was used to measure improvement in mood. It consists of 19 items, which can be scored as absent (0), mild/intermittent (1) or severe (2). The total score ranges from 0 to 38, with a higher total score indicating a greater level of depression. The measure has good internal consistency among residents with mild and moderate to severe dementia (Cronbach’s Alpha= 0.81, 0.82, respectively) [20]; this is maintained when completed by proxy (Cronbach’s Alpha= 0.86) [21].

##### Overall Adjustment

Adjustment was measured using the Index of Relocation Adjustment Scale (IRA) [22]. This consists of six items, which are measured on a 4-point Likert scale with answers ranging from completely disagree (0) to completely agree (3). The total score ranges from 0 to 18, with a higher total score denoting a greater level of adjustment. The use of the measure in this study was explorative; Hayward et al. [9] adapted the IRA to include pictures of faces ranging from very unhappy to very happy and found it to be a useful measure for residents with dementia. This brief measure was completed with residents only.

##### Feasibility of SettleIN for staff

The interviews focused on staff participants’ views on delivering the SettleIN programme. Questions included: ‘what challenges have you experienced?’ and ‘how easy or difficult has it been to finish the programme in the 4–6 weeks?’ To reduce response bias, interviews were not conducted by researchers responsible for training.

##### Feasibility Measures

To fully examine the feasibility of the enhanced version of SettleIN, the following dimensions of feasibility were measured, as recommended by Bowen and colleagues [23]: acceptability, demand, implementation, practicality and limited efficacy testing; recruitment and retention were also considered (see Table 4).

#### 2.2.6. Analysis

##### Missing Data

By proxy reports were used in the event that residents were unable to complete QOL-AD and CSDD measures. The IRA measure could not be collected by proxy. Missing data due to attrition was analysed using the last observation carried forward approach.

##### Qualitative Data

Data obtained from staff interviews was analysed using thematic analysis. Analysis was carried out using the six phases recommended by Braun and Clarke [24]. Both principal researchers coded the data individually as to ensure that the codes generated were consistent with the data set.

## 3. Results

**Figure 1:**
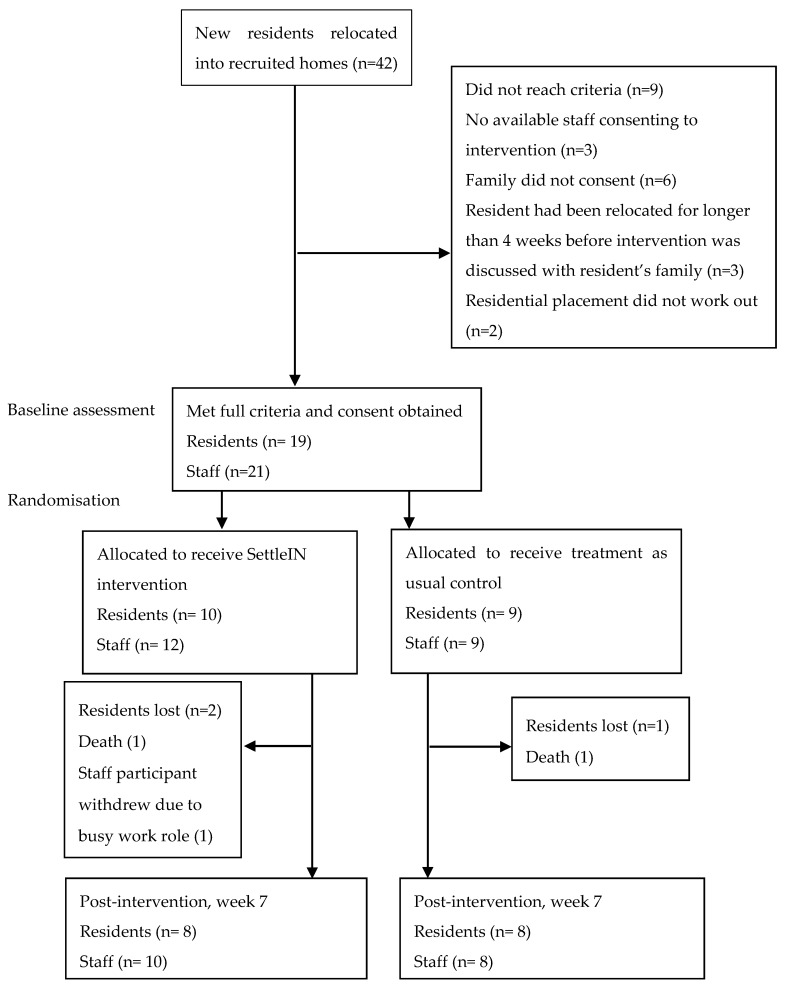
Resident participant flow chart.

Over nine months, care home managers informed the researchers of 42 new residents who had relocated into the recruited care homes (see Figure 1). From this, 19 residents from 12 care homes were eligible and took part in the study. As two of the residents had an additional staff member involved, 21 staff participants were involved in the study.

### 3.1. Resident Characteristics

The age of resident participants ranged from 73 to 96 years (see Table 5 for a summary). The majority were white British and spoke English as a first language. In total, 74 percent of residents had an Alzheimer’s disease diagnosis, as opposed to vascular or other forms of dementia. There were no significant between-group differences at baseline with regards to residents’ demographic characteristics.

### 3.2. Staff Participant Characteristics

The majority of staff were female and employed as care assistants, with the total number of years working in dementia care ranging from 9 months to 32 years (see Table 6). Their age ranged from 21 to 61 years. No significant between-group differences were found at baseline with regards to staff participants’ demographic characteristics.

### 3.3. Current Adjustment Support

All 12 care homes completed a checklist about the standard adjustment support they provided. None of the homes had a formal buddy system. Six homes showed new residents around on their first day but not as part of a continued orientation programme. Relocation assessments were used as an opportunity to learn new information about residents. Seven homes used the opportunity to ask about residents’ preferences and four used this time to ask about residents’ background information. None of the homes had special arrangements to contact family members around the adjustment period, unless there were urgent medical concerns. Five care homes also used additional methods to support adjustment including, introducing the resident to their keyworker and informing new residents of activities taking place within the home. One home also created memory boxes with new residents.

All of the residents recruited had attended a relocation assessment prior to moving into the care home. Two had a life book made, six were asked about their background information and 10 were asked about their preferences before joining the study. Notably during post-intervention interviews, staff commented that these methods were not as in depth as the SettleIN tasks.

### 3.4. Missing Data

Dementia-related impairments, physical illness or personal preference meant that ten residents (six, intervention; four, control) did not complete the Quality of Life in Alzeimer’s Disease (QOL-AD) and Cornell Scale for Depression in Dementia (CSDD) measures at baseline. All residents were approached at follow up, and nine (five, intervention; four, control) were unable to complete the measures. Nine participants (five, intervention; four, control) did not complete the IRA measure; this measure could not be collected by proxy.

Attrition-related missing data involved three resident participants (two, intervention; one, control) lost to follow up due to death and one staff withdrawal (see Figure 1 for details).

### 3.5. Exploratory Analysis of the Efficacy of the SettleIN Intervention

#### 3.5.1. Resident Psychological Wellbeing

On average, the control group experienced more depressive symptoms, as measured by the CSDD, at baseline compared to the intervention group (see Table 7). This difference was not found to be significant, *t*(17) = 1.14, *p* = 0.27.

The mean change score in the CSDD scores was compared between groups. Although a large effect size was found in favour of the intervention group (d = 0.70), independent sample t-tests indicated that this difference in mean change between groups was not statistically significant (*t*(17) = 1.45, *p* = 0.41).

#### 3.5.2. Resident Quality of Life

The change in QOL-AD scores was compared between groups. A medium effect size was found in favour of the intervention group (*d* = 0.47). However, independent sample t-tests revealed that the mean change in QOL-AD scores was not significantly different between the two groups (*t*(11.88) = 0.81, *p* = 0.43).

#### 3.5.3. Resident Overall Adjustment

At baseline, the intervention condition (n = 5) had a lower mean rating of adjustment, compared to the control condition; this difference was not significant (*t*(8) = −0.77, *p* = 0.47).

The change in IRA scores between assessment points was compared between the two groups. A large effect size was found in favour of the intervention group (*d* = 0. 91). However, independent sample t-tests indicated that the difference was not statistically significant between groups (*t*(8) = 1.28, *p* = 0.24).

### 3.6. Feasibility

#### 3.6.1. Recruitment and Retention

There was a low uptake among care homes, with one in nine of the care homes contacted consenting to partake in the intervention. Within the recruited care homes, however, there was a reasonable resident uptake; approximately 50 percent of the newly relocated residents were recruited into the trial. The study had an acceptable level of attrition; three of the 19 residents were lost to follow up.

#### 3.6.2. Implementation

All 12 staff participants in the intervention condition received one individual training session; the length of training was on average 75 min, but ranged between 60 and 90 min. The training and supervision sessions were conducted by the same principal researcher.

SettleIN workbooks were intended to provide information on programme implementation. Staff participants, however, were unable to fully complete SettleIN documentation due to their work loads and time constraints. Implementation could therefore not be assessed in this study.

#### 3.6.3. Qualitative Analysis of Staff Interview Data

Analysis of the 12 interview transcripts revealed five themes and 13 subthemes (see Table 8).

##### Organisational Barriers

Ten participants spoke about the impact of organisational barriers on programme implementation.

*Existing heavy workload.* Some described their job as “*stressful”* without the additional demands of the intervention. It seemed that implementing any programme on top of this felt like a significant addition.

“care staff are inundated and under, sort of, are under it with their work pressures and their day to day routine”.

(P3)

*Existing task-focused approach.* Participants spoke about the multiple tasks that they needed to complete as part of their job role. There was a sense that they were unable to dedicate time to a single resident as multiple residents needed their attention.

“I can’t sit in one place and only do one thing because it’s the work place”.

(P9)

It was especially difficult to implement the programme during a morning shift, as participants were preoccupied with care tasks during this time.

“if there is still someone not up, you can’t just go to do the programme, you have to keep going around”.

(P19)

*Difficult to find the time.* Many participants described their job role as ‘*busy*’. The lack of time available to do the programme meant that several participants had to work on the programme outside of work hours by coming in early, working during their breaks or working at home.

“I had to work overtime, to catch up with work I couldn’t do”.

(P17)

*Absence of managerial facilitation*. Four participants described how managerial factors prevented them from implementing the programme. They reported that their shift was frequently located on a different care floor to the resident or that they were not allocated to work with the resident participant.

“I am nearly always in the last stage of dementia, when (resident) is in the first stage… so it was a lot harder to do any of the work”.

(P19)

Staffing provisions also seemed to be a problem, as low staffing levels meant that participants had more responsibilities.

##### Programme Factors Acting as Barriers

Another theme was that elements of the programme made it less feasible to deliver.

*Documentation was challenging.* Half of participants commented on the SettleIN documentation, describing it as “*confusing”* and “*difficult”*.

“the problem is only the writing. It’s very stressful.”.

(P2A)

The documentation was perceived to be time consuming and more challenging than delivering the programme. Recommendations were made to reduce the volume of documentation or to move it on to an electronic format, a method of recording that was more familiar.

*Inflexibility of programme structure affects programme completion*. The weekly structure of the programme was seen as a barrier to programme completion. Outside factors such as annual leave, resident or staff illness meant that the programme was delayed and not completed within the four to six weeks.

“it took me two weeks to finish week one itself.”

(P11)

One participant recommended that the programme should be more flexible as to accommodate these outside influences.

##### Individual Resident Factors

All of the staff who delivered SettleIN noticed the impact of resident factors on ease of delivery.

*Dementia severity affected implementation.* The programme seemed more difficult to carry out in the context of more severe dementia. Dementia severity was perceived to affect residents’ ability to remember personal information, understand the questions asked and communicate their answer.

“I cannot assume that she does not understand, but she is not responding back, just a smile”.

(P8A)

Some felt that the programme would be easier to deliver with residents whose dementia was less severe.

“I think this is focused on the early stages of dementia”.

(P11)

In contrast to this, one participant felt that it was not the severity of the dementia that mattered, but rather the skill set of the staff.

*Resident preference affected engagement*. Five participants expressed difficulties carrying out SettleIN activities due to individual resident factors including mood, personality and physical wellbeing. On occasions, residents did not want to engage in conversation.

“It was challenging for me trying to engage with her … cos she was very ‘no no no, I don’t want to talk’”.

(P14)

##### Acceptability of SettleIN

All participants also spoke about the different feelings they had about the programme.

*SettleIN is difficult for staff.* Four participants discussed the elements of the SettleIN experience that felt testing. Two talked about having initial difficulties with the programme, struggling to understand it or feeling overwhelmed by it, which delayed implementation.

“I found it quite daunting to get it up and running.”

(P3)

There was also a perception that others would find the programme difficult in the context of their busy work role, and one participant felt that, consequently, the programme was too lengthy for a care home setting. Two participants also spoke about finding some of the conversations with residents ‘*uncomfortable’*, and one commented that the programme would be difficult for staff who were ‘*not as chatty’*.

“I didn’t feel that comfortable to ask her those kinds of things… the more personal questions.”

(P11)

*SettleIN content is acceptable to staff.* In contrast, some described the intervention as “*manageable”* and “*easy”*. Indeed, the majority spoke about their positive experiences of delivering SettleIN despite the challenges present. The programme was felt to be both “*helpful”* and “*enjoyable”*. Specifically, participants spoke about enjoying the opportunity for more in depth conversations with residents and working more closely with family members.

“It is nothing to not enjoy, because its, all the tasks, we are finding they are pleasant to do… And it is just for the benefit of knowing the person more”.

(P8A)

Participants also spoke about how much they developed during the experience. SettleIN provided them with an opportunity to be exposed to new experiences and to learn more, suggesting that there was a demand for the intervention.

“this sort of training will help people acknowledge more about dementia”.

(P6)

*SettleIN is positive for residents.* All staff participants felt that the programme had been of some benefit to the residents. The intervention helped them get to know residents more quickly and facilitated friendships with residents. Participants gave specific examples of changes they noticed in the resident as a result of the programme.

“Independence. Definitely. She’ll still come and say something, you know ‘where’s my room’ and I’ll go ‘… You show me’. And off she goes … You just stand up here with a silly grin on your face! Yeah! She’s doing this!”

(P13)

##### Overcoming Challenges

Eight participants spoke about ways in which they had attempted to overcome the feasibility issues they faced.

*External support is needed*. Half employed colleagues to support programme implementation and some relied on others to complete care tasks whilst they delivered the programme. Those who conducted the programme in pairs found this to be particularly valuable.

“If you have partner, your colleague who you can ask… they give you good ideas”.

(P8B)

Participants expressed that more support was required from the researchers for SettleIN to be fully implemented.

*Adopting problem solving.* When challenges were present, participants came up with various ways to try and solve these. Solutions included planning ahead, relying on family members, being flexible with the programme structure and using alternative means to document SettleIN conversations.

“I have no time to write it down on the paper. But I have a list … for myself”.

(P2B)

## 4. Discussions

The study explored whether an enhanced version of SettleIN improved new residents’ psychological wellbeing, quality of life and overall adjustment. In addition, it aimed to evaluate the feasibility and acceptability of SettleIN.

### 4.1. Summary of Results

#### 4.1.1. Efficacy of SettleIN

Contrary to the initial hypothesis, and despite medium to large effect sizes, the change in scores between assessment points did not differ significantly between the two conditions for any of the three outcome measures employed.

#### 4.1.2. Feasibility

SettleIN was found to be feasible with regards to staff acceptability and retention but not in terms of recruitment, wider organisational acceptability, and practicality. Most staff participants, who took part in the intervention, spoke about their satisfaction with the programme and the positive effects on residents. Organisational barriers, however, indicated that the intervention did not fit in with the wider care home culture. Organisational and individual programme factors meant that implementation could not be assessed as intended.

In the qualitative interviews, staff spoke about not having enough time to do the programme. They reported that staff shortages and difficulties getting breaks made the programme feel ‘*stressful*’ to deliver on top of other care duties. The difference in organisational support between homes perhaps contributed to the highly contrasting staff feedback, with other staff participants finding the programme both enjoyable and manageable. This highlights the significant variation between care homes and the impact that organisational factors, such as managerial support [14], can have on intervention feasibility within care settings.

#### 4.1.3. Comparison to the First SettleIN Study

The qualitative data from the first trial indicated that people found SettleIN to be too intensive in the context of organisational barriers. There were also difficulties engaging particular residents in SettleIN tasks and the reliance on family members delayed the programme. In response to the first trial, the current study reduced the length of SettleIN, included a focus on residents struggling to engage and removed dependency on family members.

Despite these changes, organisational factors remained a barrier to implementation and the impact of resident factors on programme implementation was noted. In contrast to Hayward and colleagues’ [9] findings, only one participant commented negatively on the length of the programme. This study also found evidence that SettleIN was feasible with regards to retention, disconfirming Hayward, who found high rates of attrition.

### 4.2. Limitations

This study did not manage to recruit 30 participants as desired. The small sample size likely meant that it was underpowered to detect effects. The effect size measure Cohen’s *d* was used to calculate the magnitude of difference between mean change scores of the two groups. Cohen’s *d* is a widely used and standardised effect size estimate. However, it is recognised that Cohen’s *d* is positively biased when sample sizes are small [25]. The mixed-methods design and qualitative results enabled bias to be mitigated. Despite the small sample size, credible and important information about programme feasibility, a chief aim of the study, was obtained.

Due to the methodology of the study, contamination effects may have occurred between the conditions. Each resident was recruited individually upon relocation; individual rather than cluster randomisation was therefore utilised for ethical reasons. To minimize contamination, staff in the intervention condition were instructed during training to not discuss the programme with colleagues or to use the programme with other residents.

To deal with missing data due to attrition, the last observation carried forward method was used. Although this is a widely used approach, it can introduce bias into the results [26]. The effect of the intervention may therefore have been either exaggerated or minimised for these participants.

The QOL-AD and CSDD have been shown to be valid measures for individuals with severe dementia [20,27]. However, the high proportion of residents unable to complete both measures in this study suggested that they are challenging for such individuals to complete. Staff by proxy reports were therefore relied upon. The measures selected had high levels of internal consistency for by proxies, in order to account for this. Training, however, can alter how staff perceive residents’ behaviour [28]. Staff who received training learnt about the difficulties experienced by residents and so were perhaps more likely to notice these.

Furthermore, no formal measure of adherence was included in this study, which makes it difficult to determine whether staff in the intervention condition followed the programme as intended. The study was also unable to measure implementation as planned due to challenges with SettleIN documentation. It is therefore unclear whether the full benefits of the programme were achieved. Staff qualitative feedback, however, did provide us with information about the ways in which staff completed the programme.

Phase one of the current study involved conducting a consultation with staff participants from the initial trial. Understanding the context of an intervention is key to ensuring that an intervention is deemed accessible by those delivering it [10]. Staff turnover though meant that the researchers were unable to consult with numerous participants from the previous trial. Qualitative feedback from the first trial was used alongside the consultation to account for this. However, it is likely that valuable information was lost that could have been used to improve the programme, perhaps impacting on the feasibility findings of this study.

It is possible that common systemic challenges including staffing levels and competing care priorities mean that homes do not have the resources available to support the implementation of psychosocial interventions. Attempting to evaluate SettleIN in this study was therefore problematic, and ultimately unsuccessful, within this climate.

### 4.3. Implications for Future Research

There has been little focus in dementia literature on interventions that facilitate adjustment to residential care. Sury and colleagues [3] did, however, propose various strategies that could be employed to aid the adjustment process. The findings reported do not support their suggestions that such strategies result in significant change compared to residential care as usual.

This poses a dilemma, as the qualitative feedback obtained indicated that a programme of this nature is needed whilst also suggesting that the number of barriers to programme implementation was severe. A further feasibility trial could attempt to address this. However, it would require re-thinking and re-structuring the current programme, in line with staff qualitative feedback, without losing the integrity of the evidence base that informed the design [3]. Researchers would need to return to the development phase of the Medical Research Council framework [10] to make changes to SettleIN. Despite attempts to address these barriers during the design and development stage of this study, including involving managers and reducing programme intensity, the organisational and programme-specific barriers present remained formidable. It is recognised though that trailing complex interventions can be an extensive process and several pilot studies may sometimes be needed before a full-scale trial can be conducted [10].

In order to address the programme-specific barriers found, SettleIN documentation would need to be simplified and condensed. This is in line with recent research that has found that training programmes of reduced intensity are more satisfactory to staff [13]. The structure would need to be more flexible. A possible solution would be to extend the four-week framework. This could reduce the likelihood of disruptions, which often occur in this setting, negatively affecting programme completion.

Participants spoke about the need for additional support to facilitate programme delivery. To address this, staff could be trained in pairs to help with increasing programme flexibility. A greater focus on recruitment would also be needed in order to increase the sample size and power of the study. It would also be beneficial to consider alternative ways of measuring implementation and adherence, aside from staff self-report measures.

The impact of resident factors on programme completion came up here and in the Hayward study [9]. Hence, an optional module for residents who struggle to engage was developed in response. Unfortunately, this did not fully resolve the difficulties present. The views of the resident participants were not collected during either phase of this study. Interviews with residents might have provided information about their experience of relocation and the support they would have liked to receive. Moving forward, where possible, residents should be involved during programme development stages. This is in line with Medical Research Council recommendations which highlight the importance of service user involvement during intervention development [10].

There is a need for further research to focus on the validity and accessibility of outcome measures for people with dementia. Many residents struggled to complete the measures used in this study. Creating measures that are more accessible would allow us to gain more insight about the usefulness of interventions from the perspective of the individuals that they are designed for.

### 4.4. Clinical Implications

This study highlighted the negative impact that relocation can have on residents’ psychological wellbeing, as over half of residents met criteria for depression at baseline. There is a need for adjustment support to be imbedded into care practice. Adjustment support currently offered by care homes appears minimal and often targets family members, rather than residents.

When delivering the psychosocial intervention, the majority of staff felt that they developed a stronger relationship with new residents and that the programme provided support and comfort to residents. These results point to the usefulness of staff-led psychosocial interventions for new residents and refer to factors that were perhaps missed when using quantitative outcomes. The organisational barriers present, however, showed the negative impact that heavy workloads and consequent time constraints have on care staffs’ ability to deliver psychosocial care on top of routine care tasks. These organisational issues, alongside individual programme factors, meant that SettleIN was not feasible to deliver as part of standard care, reflecting the findings of Hayward. It was hoped that making changes to SettleIN, in line with the literature, would reduce the impact of such barriers. Instead, there continues to be a challenge in fitting these strategies into everyday care.

## 5. Conclusions

Overall, the changes in resident’s quality of life, wellbeing and overall adjustment following SettleIN did not differ significantly to residential care as usual. The programme was not found to be feasible in its current format. However, qualitative data suggested that the intervention was acceptable to most staff and beneficial in some way for residents. Interviews with staff highlighted barriers to programme implementation stemming from organisational, resident and programme factors. An increased focus on reducing organisational barriers in care home research is required, so that such factors do not prevent programme implementation and change to care practice from taking place.

## Figures and Tables

**Table 1:** Summary of consultation qualitative feedback.

Theme	Feedback	Further Changes Made
Programme intensity	Reducing content made the programme more accessible Programme looked easier to do alongside job role There was too much to do in the previous version	Some activity repetitions were reduced further
Additions to the programme	New activity added would work well New activity met resident’s needs New module would be helpful for some but not all residents Supervision would be helpful	Kept new module but made it optional Agreed that supervision would be offered weekly
Individual resident factors as barriers	Resident personality and dementia severity, would influence programme feasibility and usefulness Programme dependent on resident’s verbal ability	Inclusion criteria to not include individuals with severe dementia as measured by the Functional Assessment Staging Test To meet this criteria, resident participants had to be able to speak more than 5–7 words a day

**Table 2:** Inclusion criteria.

Criteria	Setting	Residents	Staff
Inclusion	Care Quality Commission (CQC) rating of ‘requires improvement’ (that does not include safety as an improvement factor), ‘good’ or ‘outstanding’ Staffing levels to allow individual staff members leave to attend training Managerial support to participating staff	Dementia diagnosis Dementia classified as mild to moderately severe (stages 2–6) on the Functional Assessment Staging Test (FAST) Able to converse in English Relocated to the care home within the past month	Employed to support residents within the care home (may include nurses, health care assistants, care workers, team leaders, activity coordinators, etc.)

**Table 3:** Examples of an activity from each of the five SettleIN modules.

Module	Activity	Frequency	Minutes
Orientation	Introduce a buddy or buddies (at least one staff member and possibly another resident who knows their way around) to the new resident	1 time in week 1	15
Lifestyle	Gently ask the resident about how they spent their typical day, week and month prior to moving into the care home. Plan with the resident about how to keep up as many of the routines as possible (examples given)	1 time in week 2 and week 3	20
Family and Friends	Complete a simple family tree (see resources in the management manual for an example)	2 times in week 1	20
Identity	Create a ‘This is Your Life Book’ with the resident (refer to SettleIN Management Manual for guidance)	2 times in week 3 and week 4	20
Struggling to Engage (optional module)	Get to know the resident by talking to them about topics unrelated to their move. Example questions given e.g., what was your favourite holiday?	2 times in week 1	15

Note. Frequency refers to different days unless stated. Minutes refers to per attempt.

**Table 4:** Key dimensions of feasibility examined and outcomes measuring this.

Area of Feasibility	Related Research Question	How Assessed
Acceptability	Is an enhanced version of SettleIN acceptable, attractive and satisfying to stake holders?	(1) Consultation following modifications to SettleIN (2) Staff participant interview (3) Descriptive statistics of recruitment feasibility
Demand	To what extent was enhanced SettleIN used?	(1) Staff interviews
Implementation	To what extent was enhanced SettleIN successfully delivered?	(1) Analysis of SettleIN documents (2) Staff participant interview
Practicality	To what extent was enhanced SettleIN carried out with intended participants without outside intervention?	(1) Staff participant interview
Limited efficacy	Is an enhanced version of SettleIN effective in facilitating the adjustment of people with dementia who have recently been placed into residential care?	(1) QOL-AD (2) CSDD (3) IRA
Recruitment	How easy was it to recruit?	(1) Number of contacts made (2) Time taken to recruit (3) Numbers recruited
Retention	How many participants stayed in the trial?	(1) Attrition rates

Note. QOL-AD = Quality of Life in Alzheimer’s Disease; CSDD = Cornell Scale for Depression in Dementia; IRA = Index of Relocation Adjustment.

**Table 5:** Baseline resident demographic characteristics.

Characteristics	Control Condition (N = 9)	Intervention Condition (N = 10)
Age, mean (SD)	87.90 (7.20)	86.33 (6.58)
Number of days since relocation, mean (SD)	17.00 (9.30)	17.11 (7.83)
Gender, N (%)		
Female	9 (90)	7 (78)
Male	1 (10)	2 (22)
Ethnicity, N (%)		
White (British)	10 (100)	7 (78)
White (Other)	0 (0)	2 (22)
Religion, N (%)		
Church of England	3 (30)	5 (56)
Catholic	1 (10)	2 (22)
Jewish	3 (30)	0 (0)
No religion	3 (30)	2 (22)
First language, N (%)		
English	10 (100)	7 (78)
Other	0 (0)	2 (22)
Marital Status, N (%)		
Single	0 (0)	2 (22)
Married	0 (0)	1 (11)
Widowed	9 (90)	6 (67)
Divorced	1 (10)	0 (0)
Dementia diagnosis, N (%)		
Alzheimer’s disease	7 (70)	7 (78)
Vascular	3 (30)	1 (11)
Other	0 (0)	1 (11)
FAST score, N (%)		
Mild dementia	1 (10)	1 (11)
Moderate dementia	1 (10)	1 (11)
Moderately severe dementia	8 (80)	7 (78)
Number of long term health conditions, mean (SD)	4.20(1.99)	3.00 (1.58)
Number of prescribed medications taking, mean (SD)	7.70(3.68)	8.00 (5.07)

**Table 6:** Staff characteristics.

Characteristics	Intervention Condition (N = 12)	Control Condition (N = 9)
Age (years), mean (SD)	43.17 (13.72)	38.78 (12.85)
Gender, N (%)		
Female	11 (92)	7 (78)
Male	1 (8)	2 (22)
Job title, N (%)		
Care assistant/support		
worker	8 (67)	5 (56)
Senior care assistant	1 (8)	2 (22)
Team leader	1 (8)	2 (22)
Activities co-ordinator	1 (8)	0 (0)
Care manger	1 (8)	0 (0)
Years working in dementia, mean (SD)	9.88 (9.59)	7.97 (6.77)

**Table 7:** Mean pre- and post-intervention scores, mean change scores and statistical significance.

Characteristic	N	Baseline Mean (SD)	Post-Intervention Mean (SD)	Mean Change from Baseline (SD)	P	Effect Size
CSDD						
Intervention	10	10.60 (5.18)	8.20 (5.07)	+2.40 (5.52)	0.17	0.70
Control	9	13.17 (4.57)	14.83 (4.30)	−1.67 (6.69)		
QOL-AD						
Intervention	10	31.50 (5.21)	33.60 (6.17)	+2.10 (3.78)	0.43	0.47
Control	9	30.83 (4.37)	30.78 (5.65)	−0.06 (7.13)		
IRA						
Intervention	5	6.40 (2.88)	11.80 (4.67)	+5.40 (6.23)	0.24	0.91
Control	5	8.00 (3.67)	8.00 (5.05)	0.00 (7.07)		

Note. (+) = improvement; (−) = deterioration. CSDD = Cornell Scale for Depression in Dementia; QOL-AD = Quality of Life in Alzheimer’s Disease; IRA = Index of Relocation Adjustment.

**Table 8:** Themes and subthemes from staff interview data.

Themes	Subthemes
**Organisational barriers**	Existing heavy workload
	Existing task-focused approach
	Difficult to find the time
	Absence of managerial facilitation
**Programme factors acting as barriers**	Documentation was challenging
	Inflexibility of programme structure affects programme completion
**Individual resident factors**	Dementia severity affected implementation
	Resident preference affected engagement
**Acceptability of SettleIN**	SettleIN is difficult for staff
	SettleIN content is acceptable to staff
	SettleIN is positive for residents
**Overcoming challenges**	External support is needed
	Adopting problem solving
